# Use of TOFSim, a LabView-Based Time-of-Flight Mass Spectrometer Simulation, to Model Real Instrument Data

**DOI:** 10.1021/jasms.4c00406

**Published:** 2025-02-07

**Authors:** Hannah M. Palmer, Kevin G. Owens

**Affiliations:** Department of Chemistry, Drexel University, Philadelphia, Pennsylvania 19104, United States

**Keywords:** Time-of-flight mass spectrometry, simulation, MALDI, LabView, virtual instrument, flight time, peak width

## Abstract

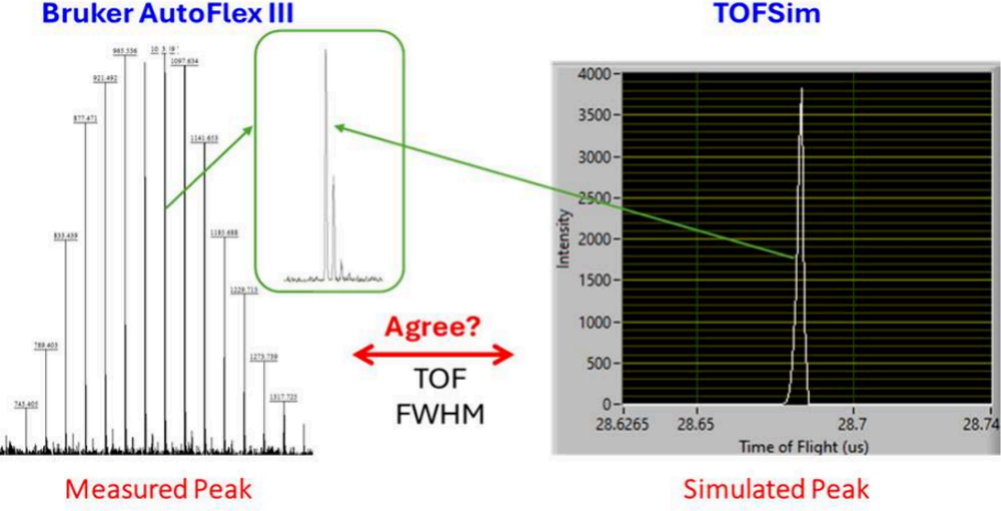

It is demonstrated here that a recently published LabView-based time-of-flight mass spectrometer (TOFMS) simulation program (named TOFSim) can accurately simulate data collected on a commercial Bruker Autoflex III matrix-assisted laser desorption/ionization (MALDI) TOFMS instrument operating in linear mode. Once the instrument distances are determined by matching measured and simulated flight times, it is shown that both overall flight times and peak widths are reproduced for data collected under both focused and slightly defocused conditions. This work confirms that TOFSim can be used not just for training new instrument operators in the principles of TOFMS but, as demonstrated here, to show how changing the voltage applied to grid G1 in the source or the delayed extraction delay time affects the focusing properties of the instrument. In the future we expect that this will also allow users to perform “what-if” experiments to investigate scenarios which may be difficult or impossible to do in a real instrument.

## Introduction

We recently published an account of a time-of-flight mass spectrometer (TOFMS) simulation named TOFSim programmed using the National Instruments, Inc. LabView graphical programming language.^1^ It can simulate both linear and reflectron-type instruments equipped with a one-step, two-step, or three-step source and a one-, two-, or three-step reflector region for a reflectron-type instrument. It has the option to model continuous extraction or delayed extraction, and is also able to simulate a selection of both gas-phase (electron ionization and multiphoton ionization) and solid-phase (matrix-assisted laser desorption/ionization, MALDI) ionization sources. The simulation allows setting the length of the flight tube as well as any of the source, reflector and detector regions. Voltages applied to the grids in the source, reflector and detector regions can be manipulated as well. These parameters can be altered in order to increase the instrument resolving power or to match those found in a real instrument. Two ions of any mass-to-charge ratio can be simulated simultaneously.

A Monte Carlo simulation is used in TOFSim to randomly generate the initial position, initial velocity, and initial time of formation of the ions created in the source. These ion formation values depend on the input mean and standard deviation values for each distribution (i.e., the position, velocity and time of ion formation) chosen by the user. Detailed flight time equations^2,3^ are used to transform these input values into a flight time for each given ion; by simulating a large collection of ions (e.g., 10,000) in each experiment, a simulated ion peak shape can be obtained. As the Monte Carlo process is used, the peak position and width (calculated from the histogram of the calculated flight time values) varies slightly each time the simulation is run, mimicking what is observed in a real experiment. Manipulation of any of the instrument parameters and ion characteristics allows the user to investigate a multitude of different experiments. TOFSim is freely available (as both editable LabView VIs and as a standalone executable) as part of the Supporting Information for ref 1.

While the initial account described how the simulation can be used for teaching the principles of operation of a TOFMS (and the publication includes an Experiment Manual containing multiple experiments designed to teach the principles of TOFMS),^1^ we have also used TOFSim in our lab to help understand data collected from a commercial MALDI TOFMS. The ability to simulate data that is representative of data collected on a physical instrument is extremely useful, as it allows the user to conduct “what-if” experiments and manipulate settings or investigate scenarios which may be difficult or impossible to do in a real instrument. TOFSim is shown here to be able to accurately simulate flight times and peak widths compared to data collected on a Bruker AutoFlex III MALDI TOFMS in the linear mode of operation. While similar work has been conducted using the program SIMION,^4^ this is the first time TOFSim has been demonstrated to be used in this manner.

One important difference between how TOFSim is designed compared to the Bruker AutoFlex III is that TOFSim models an instrument where the different flight regions are bounded by ideal metal mesh grids, whereas the Bruker instrument is of a gridless design. The openings between the grid wires, and in a gridless instrument, the openings in the metal plates defining the various instrument regions, impact how the ions experience the electric fields present due to what are known as grid effects.^5,6^ These grid effects will be explored in detail in the results presented below, and it will be shown how we can create in the simulation what we term an “equivalent gridded instrument” that can accurately reproduce both the flight times and peak widths observed on the physical instrument. In this work samples were prepared of oligomers of polyethylene glycol (PEG) over a range of molecular weight. Parameters of the simulation were adjusted to mimic the Bruker instrument, and using a series of masses chosen from the PEG data, TOFSim is shown to be able to simulate flight times and peak widths that matched the data collected on the real instrument. The simulation was also used to conduct experiments to explore the relationship between delay time and voltage applied to the first grid in the source (G1) in order to focus the instrument to obtain the highest possible resolving power.

## Experimental Section

### Materials

The MALDI matrix 2,5-dihydroxybenzoic acid (DHB, > 98%) and the synthetic polymer analyte polyethylene glycol (PEG) with peak molecular weights of 1000, 1500, and 2000 were obtained from Sigma-Aldrich (St. Louis, MO). Methanol (HPLC grade, 99.9%) was obtained from Fisher Scientific (Waltham, MA).

### Methods

#### Sample Preparation—Determining the Internal Lengths of the Bruker AutoFlex III Source and Flight Tube

PEG 1000, 1500, and 2000 samples were prepared as 0.002, 0.002, and 0.001 M solutions in methanol, respectively. A 0.09 M solution of DHB in methanol was used as the matrix for PEG 1000 and 1500 and a 0.08 M DHB solution (in methanol) was used for PEG 2000. The sample spot was made by depositing 2 μL of a mixture of 50 μL of DHB and 20 μL of PEG onto the MALDI plate using the dry drop method. The molar matrix-to-analyte ratio (M/A) for the PEG 1000 and PEG 1500 samples was 1125, and for the PEG 2000 sample it was 2000. For work in this section a single sample spot for each PEG sample was prepared and five mass spectra were collected from that spot. Ten masses were chosen for analysis from each spectrum collected from each of the PEG 1000, 1500, and 2000 samples spanning from the low end to the high end of the polymer distribution. This resulted in three mass lists composed of ten masses each.

#### Sample Preparation—Adjusting the G1 Voltage to Improve the Accuracy of Simulated Peak Width, Comparing Simulated and Measured Flight Times and Peak Widths, and Comparing Measured and Simulated Data Using the Average Values from Triplicate Measurements

A sample was prepared by mixing 2 μL of a 0.001 M PEG 2000 and 80 μL of 0.05 M DHB, both prepared in methanol, giving a M/A of about 2000; 2 μL of this mixture was spotted on three separate spots (spots M21-M23) on the Bruker MTP-384 stainless steel sample plate using the dry-drop method. For work in this section five individual mass spectra were collected from each of the three sample spots deposited on the MALDI plate. Ten masses were chosen for analysis from each spectrum collected from the PEG 2000 sample spanning from the low end to the high end of the polymer distribution. This resulted in a single mass list composed of ten masses.

### Software

All simulated data was collected using TOFSim^1^ written in LabVIEW 2018, version 18.0.1 (National Instruments Inc., Austin, TX). Data from the Bruker Autoflex was analyzed using the Bruker, Inc. FlexAnalysis version 3.4 (Build 76), GRAMS version 9.0 (Thermo Galactic, Inc., Salem, NH) and a custom-written file format conversion program named FIDtoJCAMP (version 0.6a, https://sourceforge.net/projects/fidtojcamp/).

### Instrumentation

All experimental data was collected on a Bruker (Bremen, Germany) Autoflex III MALDI TOFMS controlled using FlexControl version 3.4 (Build 135). All data was collected using the linear mode of operation.

#### Terminology

The terms used to label some of the parameters differ between the Bruker Autoflex III and TOFSim. This includes IS1, IS2, and PIE Time in the instrument, which correspond to G0, G1, and Delay Time in TOFSim, respectively.

#### Explanation of File Conversion

FlexAnalysis, the post processing software used for analyzing data collected from the Bruker instrument, can give the flight time in units of time (ns), however, it does not give the peak width measurements in units of time. In order to obtain the peak width data in units of time, a program created in the Owens lab (FIDtoJCAMP) was used to convert the native FID file created by the Bruker FlexControl software into a JCAMP format file. Once the files were in JCAMP format, the GRAMS v9 (Thermo Galactic, Inc.) software was used to open each file giving a spectrum with the *x* axis in time and the *y* axis in intensity. The integration tool in GRAMS was then used for manual peak integration for about 20 peaks per spectrum. This data was then compared to the original data obtained on the Bruker instrument to compare flight times from the FlexAnalysis peak picking and the manually integrated peaks in GRAMS.

#### Instrument Parameters—Determining the Internal Lengths of the Bruker AutoFlex III Source and Flight Tube

The data in this section was collected using the following parameters: IS1 set to 20.1 kV, IS2 to 18.64 kV, the lens was 7.5 kV, the PIE delay was 0 ns (note that the Bruker instrument has a built-in delay time of 180 ns) and the detector setting was −1500 V.

The data sets for the sections described below were collected using two different instrument methods in order to develop a larger sample set to test TOFSim against. The two methods were: 1) one tuned that day to achieve the highest resolving power, and 2) one that was purposely not tuned. These methods are referred to as the LA method and LB method, respectively (where L stands for “linear”).

#### Instrument Parameters—Adjusting the G1 Voltage to Improve the Accuracy of Simulated Peak Widths, Comparing Simulated and Measured Flight Times and Peak Widths, and Comparing Measured and Simulated Data Using the Average Values from Triplicate Measurements

For the LA method, IS2 was set to 19.0 kV, the lens was 7.6 kV, the PIE delay was 100 ns and the detector was −1536 V. In the LB method, IS2 was set to 18.9 kV, the lens was 7.56 kV, the PIE delay was 60 ns and the detector was −1661 V. IS1 was set to 20.1 kV for both methods.

### Simulation Parameters

TOFSim was designed to accurately mimic TOF data; the user can change many instrument parameters to best recreate the setup of the instrument that is being used for experimental work. For the section Determining the Internal Lengths of the Bruker AutoFlex III Source and Flight Tube, the IS1, IS2, detector voltage, and PIE time were all set to match exactly as they were recorded from the instrument at the time of data collection. It should be noted that at the present time there is no lens included in the simulation. In the section Adjusting the G1 Voltage to Improve the Accuracy of Simulated Peak Widths, IS2 (G1) is adjusted in order to find the best agreement between the experimental and simulated peak widths.

Some parameters in the simulation have been kept constant throughout all the experiments, including, the initial position mean and standard deviation, initial velocity mean and standard deviation, initial time mean and standard deviation, linear detector acceleration distance, linear detector internal region distance, and the potential in the field free drift region (all values provided in Table 1). Note that the initial position of the ions in a MALDI experiment depends mostly on the size of the matrix crystals that form in a nonuniform pattern on the surface of the MALDI sample during the dried-drop preparation used here. As we are using the highly volatile methanol as a solvent, which is a good solvent for both the matrix and analyte (i.e., both entities have high solubility in this solvent), as well as low matrix concentrations, the size of the sample crystals produced is small (the mean distance of 1um is obtained by optical microscopy measurements of samples made in our lab). As the laser intensity is kept low for these experiments, we estimate that we ablate material over a very small range of distances (the small standard deviation of 0.2 μm) the during the MALDI experiment. The initial velocity of ions produced during a MALDI experiment has been investigated,^7,8^ and has been found to be dependent on the matrix chosen and the laser intensity illuminating the sample. The average velocity of 1000 m/s comes from these references, while an estimate of the standard deviation was obtained from previous work in our lab.^5^ As the temporal duration of the MALDI desorption laser pulse on the Bruker Autoflex is small (∼3 ns), the initial time mean and standard deviation were set to zero. Due to the lower resolution obtained from these linear TOF experiments, these variables were not investigated further in this work.

**Table 1:** TOFSim Parameters Kept Constant Throughout All Experiments

Ion Distributions	
Initial position (average):	0.001 mm
Initial position (SD):	0.0002 mm
Initial velocity (average):	1000 m/s
Initial velocity (SD):	200 m/s
Initial time (average):	0 ns
Initial time (SD):	0 ns


Detector	
Delay channels:	10,000
Data channels:	50,000
Time resolution:	1 ns


Distances	
dl:	2.20 mm
d2:	12.0 mm
d7:	2 mm
d8:	2 mm
L1:	1.26 m

All simulated data was taken using the linear instrument setup, using MALDI ionization, and with a two-step source. The distances in the two steps of the source and the flight tube vary as they were further optimized as described in the Supporting Information section titled Using Simplex Optimization to Determine the Lengths in a TOFMS.

## Results and Discussion

### Determining the Internal Lengths of the Bruker AutoFlex III Source and Flight Tube

**Figure 1:**
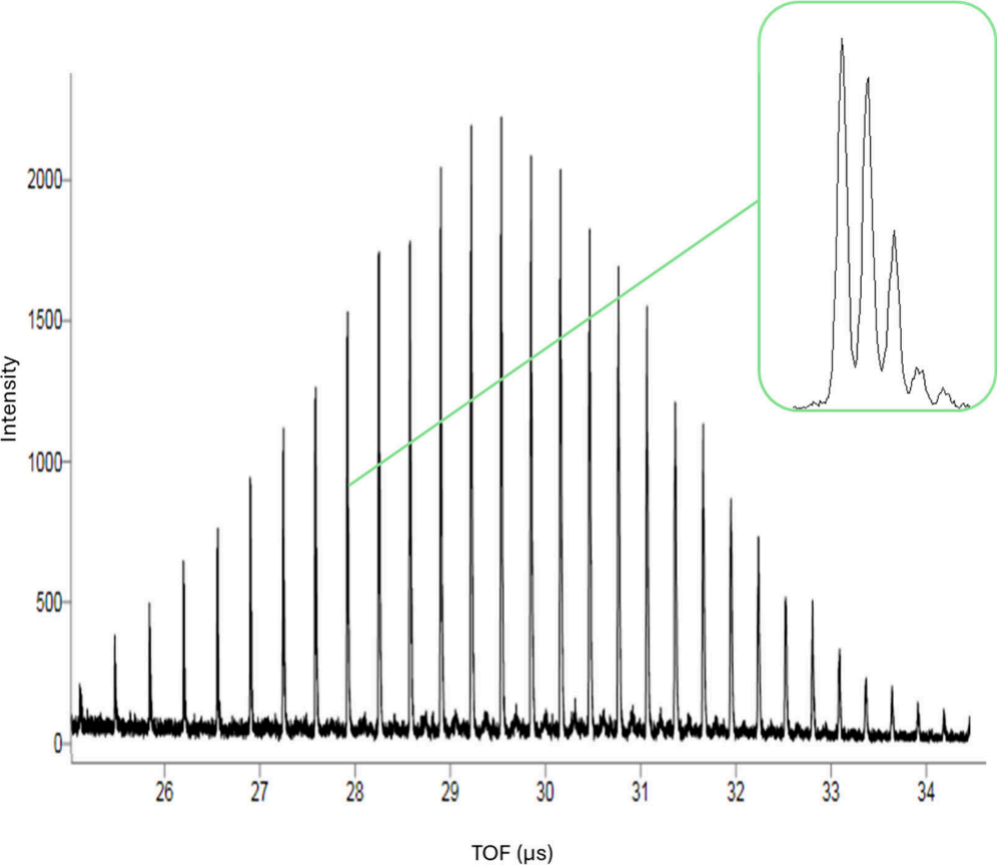
Mass spectrum of a PEG 2000 distribution collected using method LA (spectrum 2, collected on spot M22) shown with the *x*-axis in time-of-flight. Numerical values of the mass and flight time are provided in Table S1. Note that predominantly the main, sodium cationized distribution is observed.

Figure 1 shows an example of the results obtained for the analysis of a PEG 2000 sample (an additional example of results from the analysis of the PEG 1000, PEG 1500 and PEG 2000 samples is shown in Figure S2 in the Supporting Information). Table S1 in the Supporting Information gives the flight time (in us) of the monoisotopic peak of the sodium cationized species of ten of the oligomer clusters observed in this spectrum. An expansion of the peak at 1802 *m*/*z* is shown in the inset of the figure to illustrate the resolving power obtained for this analysis. Figure S3 in the Supporting Information shows a comparison of the measured oligomer monoisotopic peak in the expansion to a simulated peak of the same *m*/*z* value.

The ability of TOFSim to accurately simulate flight times for data collected on an instrument had not been previously investigated. It was hypothesized that if the parameters taken from the instrument method used by FlexControl were applied to TOFSim, it should be able to generate simulated flight times that were accurate when compared to the measured flight times. Parameters such as voltages and delay time were able to be read from FlexControl and applied to TOFSim, however, the internal lengths of the Bruker AutoFlex III instrument source and flight tube were not known and not available as public knowledge. Since the flight time is literally defined as the time it takes for an ion to move from the source to the detector, accurately determining these lengths was critical.

It was decided that three areas within the Bruker AutoFlex III would be the focus of these experiments because this is where an ion spends the majority of its time. As shown in Figure S1 in the Supporting Information, these areas included the first (d1) and second (d2) step in the source and the flight tube length (L). Disassembling the Autoflex to measure these distances was not an option, but an estimation of the lengths of these areas needed to be made in order to provide a reasonable starting point for the simulation. The flight tube length could be estimated by opening the outer case of the instrument and using a meter stick to take a rough measurement. While the source could not be observed directly, a spare source was available from an old instrument. These measurements gave estimated values of 1.25 m for L, 2.0 mm for d1, and 12.0 mm for d2. The detector regions d4 and d5 were kept at the default values of 2.00 mm in the simulation and were not carried through the optimization because the amount of time spent in those regions by an ion is negligible.

The accuracy of the simulated flight time and fwhm peak width was tested by setting the parameters used in the simulation to those used on the Bruker instrument for the ion masses gathered from the PEG 2000 distribution. The masses chosen from the PEG sample were the masses with the highest signal-to-noise ratio that had high enough resolution to be able to be manually integrated. A simplex optimization was performed as described in the Supporting Information section titled *Using Simplex Optimization to Determine the Lengths in a TOFMS*. The second trial of the simplex optimization yielded values for d1, d2, and L of 2.00 mm, 12.00 mm, and 1.26 m, respectively. These values were then used as the settings in the TOFSim program to simulate data presented in the Comparing Simulated and Measured Flight Times and Peak Widths section below.

### Comparing Simulated and Measured Flight Times and Peak Widths

Examples of the data collected are shown in Table 2 for peaks from mass spectrum 2 (of five collected on sample spot M22) for the PEG 2000 sample collected using the LA method. Note that masses given in all tables are the theoretical mass of the sodium cationized monoisotopic peak; the measured flight time, simulated flight time, difference between the two, and the root-mean-square (RMS) error are all given in microseconds (us). The RMS error is found to be 0.100 us, corresponding to an accuracy of ∼3.4 parts per thousand at *m*/*z*= 1978, approximately the center of the distribution. The flight times show the expected increase with increasing *m*/*z* value; similar flight time accuracies were found for data from each of the three PEG distributions studied.

**Table 2:** Measured and Simulated Flight Times for the LA Method Spectrum 2 (Collected on Spot M22)

	TOF (μs)		
Mass	Measured	Simulated	Difference
1581.917	26.231	26.316	0.085
1625.944	26.590	26.679	0.089
1669.970	26.951	27.036	0.085
1713.996	27.300	27.389	0.089
1758.022	27.637	27.738	0.101
1802.048	27.977	28.083	0.106
1846.075	28.322	28.423	0.101
1890.101	28.652	28.759	0.107
1934.127	28.981	29.092	0.111
1978.153	29.298	29.420	0.122

RMS (μs):			0.100

**Figure 2:**
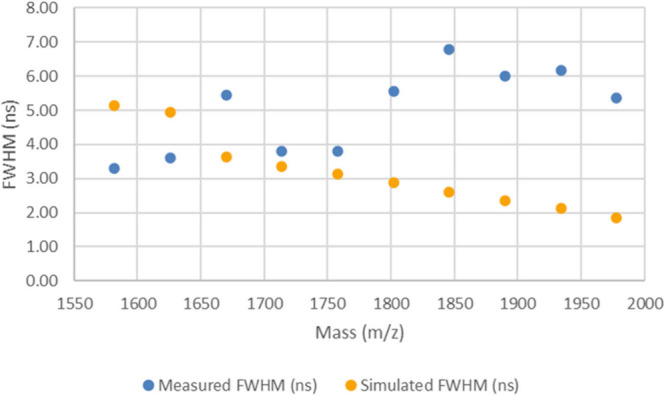
Plot of measured and simulated peak widths for the LA method spectrum 2 (from spot M22).

Figure 2 shows the measured and simulated full-width at half-maximum (fwhm) peak widths, again obtained from spectrum 2 collected using method LA on spot M22. Numerical values for the peak widths are provided in Table S4 in the Supporting Information. Method LA (the tuned method) was expected to show the best agreement due to this instrument method producing spectra with the highest resolution of the two methods, which yielded narrower peaks with greater signal-to-noise (S/N) ratio. Note the decreasing trend in the simulated peak widths (shown by the yellow data points); this suggests that the simulated instrument is focused at a mass slightly above that shown in the plot. The focusing properties of the instrument will be explored in more detail in the section titled Using Simulated Data to Explore Trends in Peak Width.

Table 3 shows that the RMS error of the flight times for data collected using the LB method is better than that found for the LA method (as shown in Table 2). This corresponds to a flight time accuracy of ∼1.3 parts per thousand at *m*/*z*= 1978. The main difference in instrument parameters that could be the reason for the improved flight time agreement is the difference in the delay time (the time between creation of ions by the desorption/ionization laser and application of the extraction voltage pulse to accelerate the ions out of the source). This cannot be determined within the scope of these experiments, however, due to the other variables not being held constant when the work was performed.

**Table 3:** Measured and Simulated Flight Times for the LB Method Spectrum 2 (Collected from Spot M21)

	TOF (μs)		
Mass	Measured	Simulated	Difference
1581.917	26.220	26.289	0.069
1625.944	26.579	26.652	0.074
1669.970	26.932	27.010	0.078
1713.996	27.281	27.363	0.083
1758.022	27.625	27.712	0.087
1802.048	27.965	28.057	0.092
1846.075	28.301	28.397	0.096
1890.101	28.633	28.733	0.100
1934.127	28.961	29.065	0.104
1978.153	29.285	29.393	0.108

RMS (μs):			0.090

**Figure 3:**
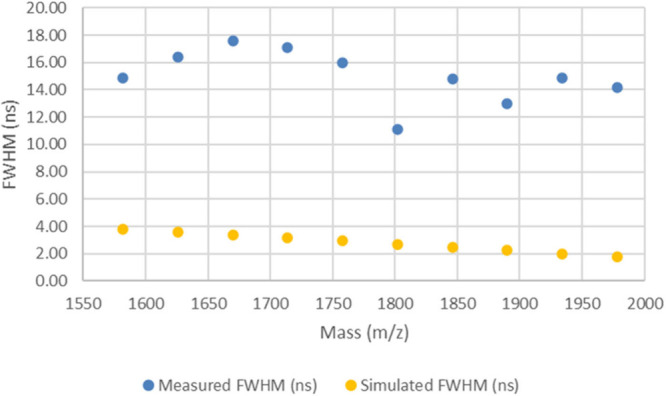
Plot of measured and simulated peak widths for the LB method spectrum 2 (collected from spot M21).

While the flight time accuracy for method LB is slightly better than that of method LA, as shown by the peak width data plotted in Figure 3, the measured peak widths for the LB method are observed to be 4–5 times wider than those of the simulated data (numerical values of the peak widths are provided in Table S5 in the Supporting Information). This was not unexpected, as the LB method was purposely left detuned; it was observed that these settings on the instrument gave spectra with very irregular broadened peaks.

While the simulated flight times showed good agreement with the measured values in both cases, the simulated peak widths, specifically for the detuned method LB, show poor agreement with the measured values. As the simulated peak widths were significantly narrower, this suggests that the actual conditions experienced by the ions in the instrument are different than those estimated from the experimental settings (and as modeled using TOFSim). As noted in the introduction, the Bruker Autoflex III is of a gridless design, while TOFSIM is designed with the presence of ideal metal mesh grids, therefore, we investigated if the differences observed might be explained by the presence of grid effects.^5,6^ If present, these grid effects would affect the electric field experienced by the ions within the source region of the Bruker instrument.

### Adjusting the G1 Voltage to Improve the Accuracy of Simulated Peak Widths

The results shown in Figures 2 and 3 (and the numerical values provided in Tables S4 and S5 in the Supporting Information) suggested that an adjustment needs to be made in one or more of the parameters of TOFSim. For a linear TOFMS, it is known that small changes in the source voltages (G0 and G1) significantly affect the focusing properties of the instrument—which affects the observed width of the ion peaks at the detector. This is due to the effect of the G0 and G1 voltages on the instrument space-focus, as first described by Wiley and McLaren,^9^ as well as the effect of the magnitude of the “delayed extraction” pulse voltage, which is given by the difference between the G0 and G1 voltage as described by Colby and Reilly.^10^ Slight changes to the voltage applied to G1 were investigated to determine if they would result in a decrease in the RMS error and improved overlap of the simulated and measured peak widths when plotted. This disagreement between the simulated and measured peak widths with the set G1 voltages was hypothesized to be due to grid effects. The “grid effect” refers to how an ion experiences the actual electric fields located between the metal wires composing the grids in the instrument, in this case in the source. The magnitude of the electric potential experienced by an ion moving between the grid wires is lower than that at the surface of the wire; the experienced potential depends on the distance of the ion from the metal wire, and for ions in a real source, there will be a distribution of those experienced potentials. The specific field felt by the ion affects the velocity at which the ion moves through the area. The grid effect is even more important in a gridless instrument like the Bruker Autoflex III, because the size of the holes in the source plates where the ions pass is even larger than the spacing of grid wires in instrument sources that include metal mesh grids. This effect is not modeled in TOFSim, so it may have been the source of the disagreement between the measured and simulated data. The difference would have a small effect on the overall flight time, but it could significantly impact the observed peak width. Since it is much easier to adjust the voltages in TOFSim, it was determined that the G1 value in the simulation would be the parameter that was adjusted. The method for determining the adjusted G1 voltage is provided in the Supporting Information; the adjusted values that provided the best agreement between the measured and simulated peak widths are given in Table S7 in the Supporting Information.

The data in Table 4 shows that the RMS error in flight time for the LA (tuned) method did not change significantly when compared to the data shown in Table 2, which was expected as changing the G1 voltage by the small values found here should have a minimal impact on the overall flight time of the ion.

**Table 4:** Measured and Simulated Flight Times for the LA Method Spectrum 2 (Collected from Spot M22) Using the New G1 Voltage

	TOF (μs)		
Mass	Measured	Simulated	Difference
1581.917302	26.231	26.335	0.104
1625.943517	26.590	26.698	0.108
1669.969732	26.951	27.056	0.105
1713.995946	27.300	27.409	0.109
1758.022161	27.637	27.758	0.121
1802.048376	27.977	28.103	0.126
1846.074591	28.322	28.443	0.121
1890.100805	28.652	28.780	0.128
1934.12702	28.981	29.113	0.132
1978.153235	29.298	29.441	0.143

RMS (μs):			0.120

**Figure 4:**
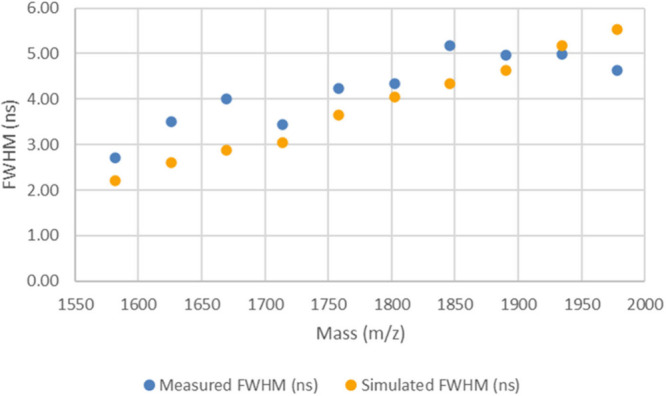
Plot of measured and simulated peak widths for the LA method spectrum 2 using the new G1 voltage (numerical values in Table 5).

However, the RMS error in peak width shown in Table S8 in the Supporting Information for the tuned method LA is now 4X less than the RMS error reported in Table S4, demonstrating that adjusting the G1 voltage significantly improved the agreement between the measured and simulated data. This is also shown by the better overlap of the two sets of data shown in Figure 4. It should also be noted that the measured and simulated data show a similar trend of increasing peak width with increasing mass.

It was mentioned earlier that the data set from method LB gave the smallest RMS error in flight time (Table 3), and it is shown to have remained the most accurate after the G1 voltage adjustment by the RMS error value reported in Table 5.

**Table 5:** Measured and Simulated Flight Times for the LB Method Spectrum 2 (Collected on Spot M21) Using the New G1 Voltage

	TOF (μs)		
Mass	Measured	Simulated	Difference
1581.917	26.220	26.289	0.069
1625.944	26.579	26.652	0.074
1669.970	26.932	27.010	0.078
1713.996	27.281	27.363	0.083
1758.022	27.625	27.712	0.087
1802.048	27.965	28.056	0.092
1846.075	28.301	28.396	0.096
1890.101	28.633	28.733	0.100
1934.127	28.961	29.065	0.104
1978.153	29.285	29.393	0.108

RMS (μs):			0.090

**Figure 5:**
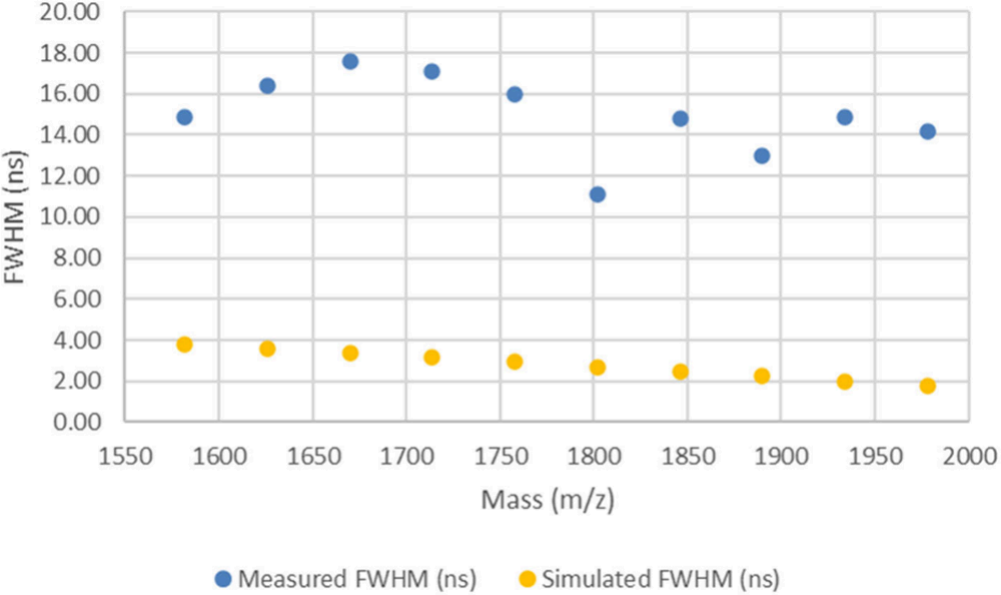
Plot of measured and simulated peak widths for the LB method spectrum 2 (collected on spot M21) using the new G1 voltage.

However, adjustment of the G1 value leads to significantly better agreement in the measured versus simulated peak widths for the LB method as shown in Figure 5. The improvement is shown numerically by the RMS error of 1.98 ns in Table S9 in the Supporting Information, which is approximately six times smaller than the RMS error value (12.30 ns) observed using the original G1 value as shown in Table S5. Figure 5 shows that there is much better agreement between the measured and simulated data; further, it appears that the slight downward trend in the measured data is now reflected in the simulated data.

### Statistical Analysis of Replicated Measured Data

**Figure 6:**
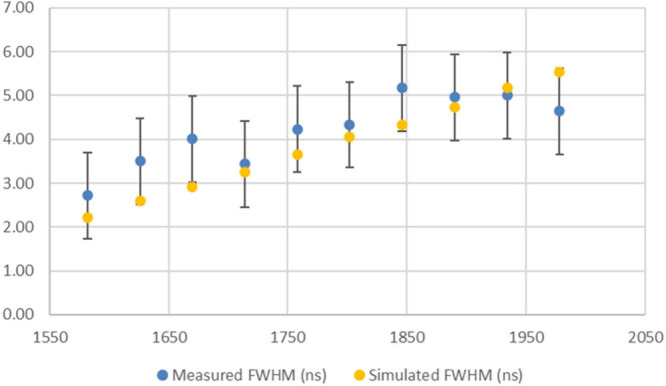
Plot of measured vs simulated peak widths for the LA method spectrum 2 (taken on spot M22), where the peak widths were measured three times. Note that similar results are found for the detuned LB method, as shown in Tables S10 and S11 and Figure S4 in the Supporting Information.

Early work in this project relied on single measurements of the peak positions and peak widths using the GRAMS program. It was noticed that there was variability in the measured peak widths that depended on manually setting the peak integration limits in the program. To investigate this, each spectrum was measured three different times, and the average and standard deviation values are used to understand the variability in the data measurement step. This allowed for the determination of error bars, which are included on Figure 6 in this section. The examples shown in Tables 6 and 7 are the data sets which gave the best agreement (the lowest RMS error values) in measured and simulated peak width. The flight times of the masses obtained using the LA (tuned) method shown in Table 6 are consistent when compared to earlier data in Table 4.

**Table 6:** Example of Measured vs Simulated Flight Times for the LA Method Spectrum 2 (Taken on Spot M22), Where the Flight Times Were Measured Three Times

	TOF (μs)		
Mass	Measured	Simulated	Difference
1581.917	26.231	26.334	–0.103
1625.944	26.590	26.697	–0.108
1669.970	26.943	27.055	–0.112
1713.996	27.293	27.408	–0.115
1758.022	27.637	27.758	–0.121
1802.048	27.977	28.102	–0.125
1846.075	28.313	28.442	–0.129
1890.101	28.645	28.779	–0.134
1934.127	28.974	29.112	–0.138
1978.153	29.298	29.441	–0.143

RMS (μs):			0.123

**Table 7:** Example of Measured vs Simulated Peak Widths for the LA Method Spectrum 2 (Taken on Spot M22), Where the Peak Widths Were Measured Three Times

	fwhm (ns)		
Mass	Measured	Simulated	Difference
1581.917	2.72	2.21	0.51
1625.944	3.50	2.60	0.91
1669.970	4.01	2.92	1.09
1713.996	3.44	3.25	0.19
1758.022	4.23	3.65	0.58
1802.048	4.33	4.05	0.28
1846.075	5.17	4.33	0.83
1890.101	4.96	4.73	0.23
1934.127	5.00	5.18	–0.18
1978.153	4.64	5.53	–0.89

RMS (ns):			0.66

The peak width agreement improved when using the average peak widths, as observed by the RMS error value in Table 7 compared to the one in Table S8. This is likely due to the measured values in Table 7 being more uniform as they are the average of triplicate measurements, which is also reflected in the error bars (representing ±1 standard deviation) shown for the data plotted in Figure 6. Note that the simulated peak widths fall within the error bars at most masses.

### Using Simulated Data to Explore Trends in Peak Width

**Figure 7:**
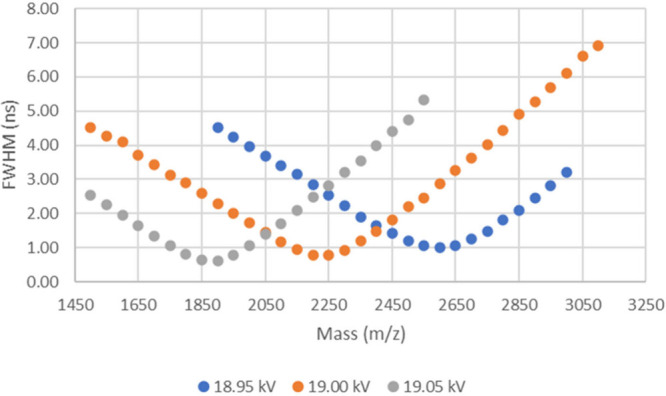
Peak width curves generated from simulating a range of masses at different G1 voltages at a fixed delay time of 60 ns, showing the change in peak width as mass increases.

The power of using the simulation TOFSim can be demonstrated in understanding the observed trends in measured peak width. It was particularly apparent after making changes in the G1 value to improve agreement between the measured and simulated peak widths that there are definitive increasing or decreasing trends to the peak widths with *m*/*z* value observed in the mass spectra. Since TOFSim can simulate two masses simultaneously in a very short period of time (approximately 1 s), it was used to investigate these trends. A range of masses from 1500–3100 Da were simulated using different G1 values, randomly chosen but evenly spaced, at a fixed delayed extraction delay time in order to determine what type of trend was occurring in the peak widths. All simulation parameters were kept constant as described in Table 1 (note that the original lengths for d1, d2, and L were used due to the timing of this work) and the G0 voltage was 20.1 kV, the drift voltage was 0 V, the detector voltage was −1500 V, and delay time was 60 ns. The G1 voltages used are shown in the legend of the plot in Figure 7. Figure 7 demonstrates a trend in focusing of the TOFMS at higher masses with lower G1 voltages (as evidenced by narrower peak widths) which corresponds to larger delayed extraction pulse voltages (as the pulse voltage is given by the difference between the set G0 and G1 voltages) at a fixed delay time.

**Figure 8:**
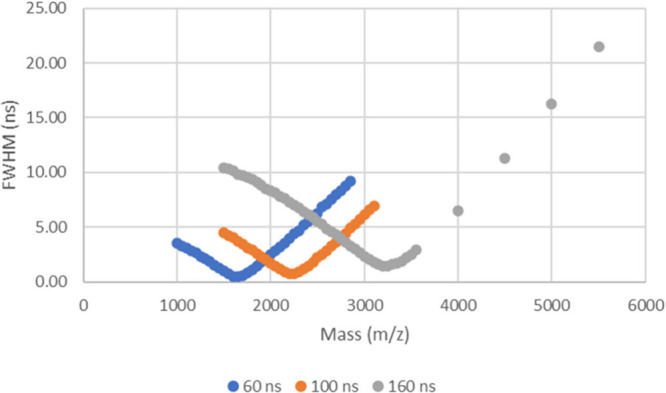
Curves generated from simulating a range of masses at different delay times, showing the change in peak width as mass increases.

A similar simulated experiment was conducted to investigate the change in peak width across a larger mass range using different delay times. The same TOFSim parameters were used except the G0 voltage remained constant at 20.1 kV, the G1 voltage at 19.0 kV, the drift voltage at 0 V and the delay times used are shown in the legend in Figure 8. This showed that shorter delay times give narrower peaks at lower masses compared to the longer delay time which showed the narrowest peaks at a higher mass (i.e., the focus of the TOFMS shifts to higher mass with delay time at a fixed delayed extraction pulse voltage).

The G1 voltage (which for a fixed G0 voltage determines the delayed extraction pulse voltage) and the delayed extraction delay times were adjusted due to those two being the factors that determine at what *m*/*z* value the TOFMS instrument is focused. The data plotted in Figures 7 and 8 are examples of focusing curves, where the focal point of the instrument occurs at the *m*/*z* value showing the narrowest peak width. Any change to the G1 voltage or the delayed extraction delay time will move this focal point as demonstrated by the three different curves in Figures 7 and 8. It should be noted that the narrowest peak width measured from the Bruker Autoflex III instrument data is 2.72 ns (Table 7) from the triplicate data with an adjusted G1 voltage, but the simulated focal point peak widths shown for example in Figure 7 are approximately 1 ns. This suggests that there may be one or more values related to the initial distributions of the ions used in TOFSim (i.e., the initial position, velocity or time of ionization) that do not match with what is actually experienced by the ions formed in the Bruker instrument. Investigating changes in these variables requires the analysis of higher resolving power data, obtained in a reflectron TOFMS experiment, which will be the focus of a future publication.

## Conclusions

The data presented here demonstrate that the LabView-based simulation TOFSim is capable of accurately simulating both ion flight times and peak widths- showing that TOFSim can be used as a reliable tool to simulate ion behavior in a time-of-flight mass spectrometer. The distances in the two-step source and flight tube have been shown as the primary parameters for obtaining accurate flight times, while adjustments to the G1 voltage were shown to result in better simulation of the measured peak widths. In this work we were able to create a “gridded equivalent instrument” which could be used to simulate data obtained in the linear mode of operation of the gridless Bruker Autoflex III instrument. The simulation was then used to create “focusing curves” that demonstrate how the choice of G1 voltage and delayed extraction delay time affect the *m*/*z* value where the instrument produces the best results. This can be used as a guide to setting up the instrument for different experiments, or in training new operators in the principles of TOFMS. Future work will focus on extending this work to data collected from operation of the instrument in reflectron mode.

## Supporting Information Available

Additional figures and tables of data as described in the text (PDF)
