# Supplementary material for: Use of TOFSim, a LabView-Based Time-of-Flight Mass Spectrometer Simulation, to Model Real Instrument Data
Source: J Am Soc Mass Spectrom. 2025 Feb 7;36(3):534–41. doi: 10.1021/jasms.4c00406 (PMC11887436; doi:10.1021/jasms.4c00406)
Supplement: Supplementary file 1 — js4c00406_si_001.pdf [file js4c00406_si_001.pdf]

Supporting Information

Use of TOFSim- a LabView based Time-of-Flight Mass Spectrometer Simulation- to Model Real Instrument Data

Hannah Palmer, Kevin G. Owens*
Department of Chemistry, Drexel University, Philadelphia, PA 19104 USA
***** Corresponding author:
kevin.owens@drexel.edu

Determining the Internal Lengths of the Bruker AutoFlex III Source and Flight Tube

Table S1: List of ten masses from the PEG 2000 spectrum (spectrum shown in Figure 1) and the measured flight times.

Mass	Measured TOF (µs)
1581.917	26.234
1625.944	26.592
1669.970	26.946
1713.996	27.294
1758.022	27.639
1802.048	27.979
1846.075	28.314
1890.101	28.646
1934.127	28.975
1978.153	29.299

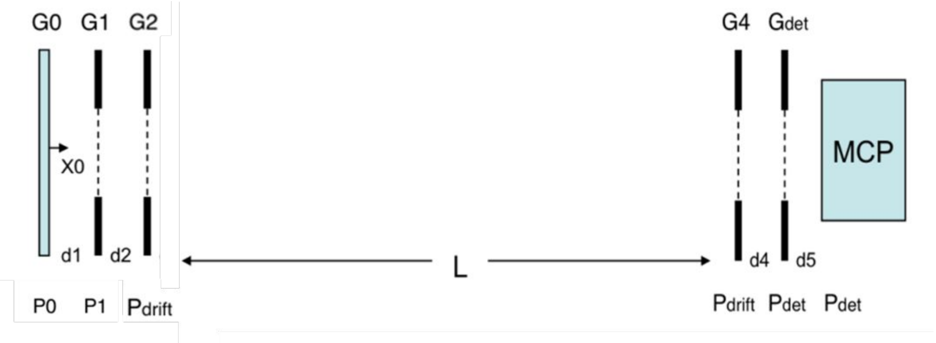

Figure S1: Diagram of a gridded two-step source (d1 & d2) linear TOFMS with a post-acceleration region (d4) and a microchannel plate detector (MCP), as modeled by the TOFSim program. Voltages P0 and P1 are applied to grids G0 and G1 (corresponding to voltages IS1 and IS2 in the Bruker instrument); Pdrift is the voltage on the instrument drift tube (0 volts in the Bruker Autoflex III, as the flight tube is grounded) and Pdet is the voltage applied to the detector. Grids G2 and G4 define the ends of the field free drift tube in the TOFSim model. Note that ions are created a distance x0 above the surface of plate G0 with an initial velocity of v0 at a time of ionization t0.

Using Simplex Optimization to Determine the Lengths in a TOFMS

The lengths obtained from the measurements taken manually were unfortunately not accurate enough to be used in TOFSim but were used as starting points in a simplex optimization program developed in the Owens Research Group called TOFSIM/PeaksMS. This program was developed from an earlier Probability Density Function (PDF) based TOFMS simulation program, which was updated to include the simplex optimization function and to replace the PDF methodology with the Monte Carlo approach[S1] also used in TOFSim. This code was used as the starting point when TOFSim was developed in LabVIEW but not all functions (including the simplex optimization routine) were transferred.

The PeaksMS program uses a list of ten masses coupled with the respective flight times obtained from the Bruker instrument (such as those provided in Tables S1). The TOFSIM/PeaksMS program uses the variable parameters for a set number of iterations until the response value (which is the sum of the differences between the ten measured and simulated flight times squared) reaches a set convergence point (in this work the convergence was set at 100 iterations of the simplex routine). The optimum values can be local or global optima depending on the chosen step size, high and low values, and initial value (the restraints used in the optimization). In an attempt to find a global optimum, the simplex optimization was run multiple times, each time making slight adjustments to the step size, high and low values, and initial value, in the direction the last optimized values were headed. The starting points for the first and second optimization trials are shown in Supplemental Tables S2 and S3, respectively.

Table S2: Starting and restraint values for the instrument parameters being optimized in the first optimization trial.

Name	Set	High	Low	Step
D1 (m)	0.002	0.004	0.001	0.0005
D2 (m)	0.01	0.014	0.009	0.003
L (m)	1.2	1.4	1.1	0.01

Table S3: Starting and restraint values for the instrument parameters being optimized in the second optimization trial.

Name	Set	High	Low	Step
D1 (m)	0.002	0.01	0.001	0.0005
D2 (m)	0.01	0.014	0.009	0.003
L (m)	1.2	1.5	1	0.1

The second trial of the simplex optimization gave values for d1, d2, and L of 2.00 mm, 12.00 mm, and 1.26 m, respectively. These values were then used as the distances in TOFSim and ten masses from the PEG 1000, 1500 and 2000 distributions (mass spectra shown in Figure S2) were used to generate simulated flight times that were compared to the flight times recorded from the physical instrument. These results are discussed in the main body of the article in the section titled *Comparing Simulated and Measured Flight Times and Peak Widths*.

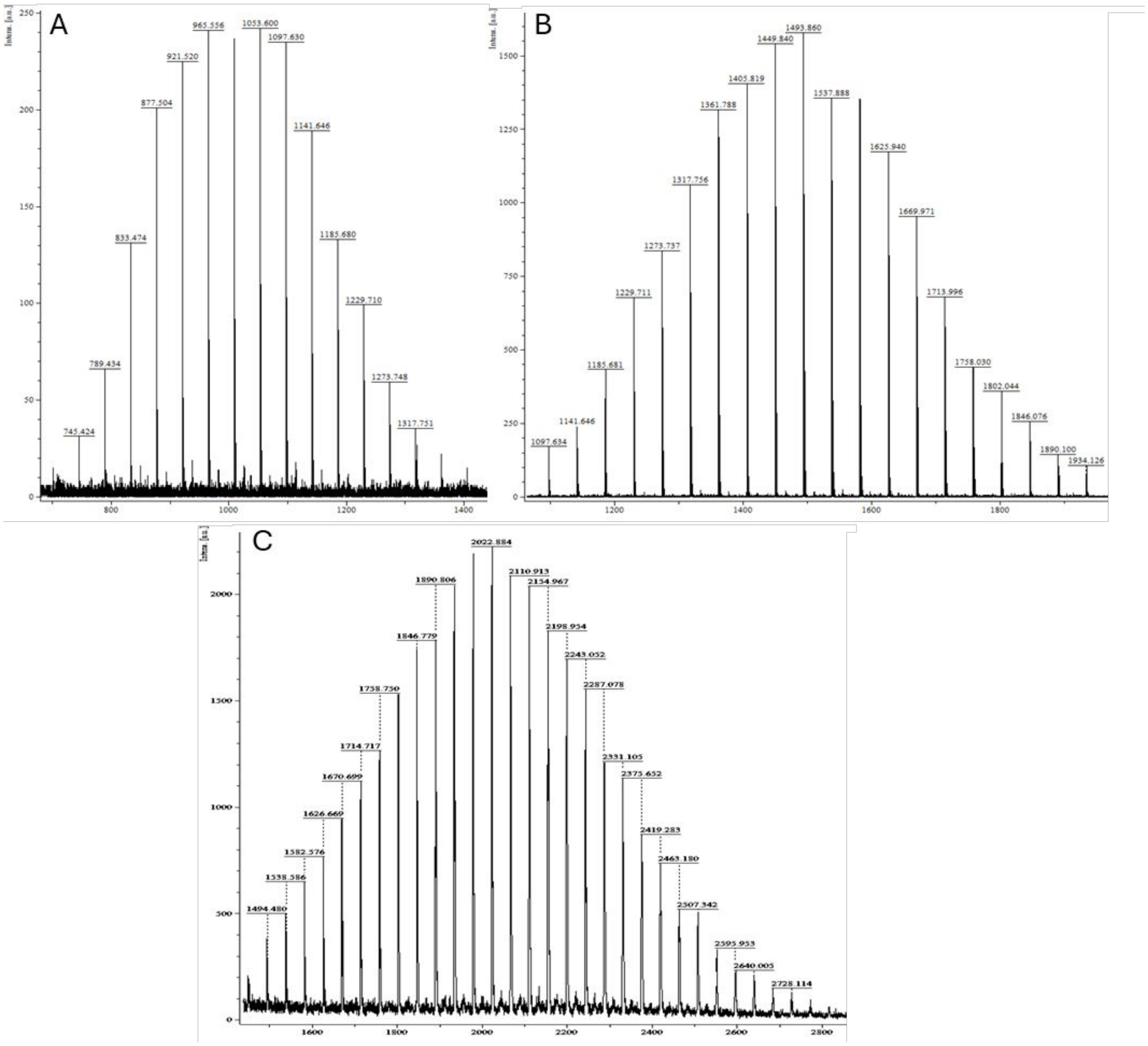

Figure S2: Examples of mass spectra collected using method LA from a PEG 1000 (A), PEG 1500 (B) and PEG2000 sample (C).

Comparing Simulated and Measured Flight Times and Peak Widths

Figure S3 shows a comparison of a measured isotope cluster and a peak simulated using TOFSim for the monoisotopic mass in that cluster.

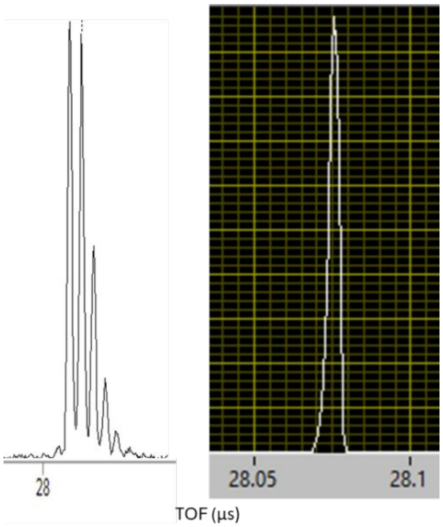

Figure S3: Comparison of a measured and simulated peak at m/z = 1802.048.

Table S4 and S5 show a comparison of the measured and simulated peak widths collected from the PEG2000 sample using the tuned (LA method) for spectrum two (taken at spot M22) and detuned (LB method) for spectrum two (taken at spot M21), respectively. Plots of this data are in Figures 2 and 3, respectively, in the main document.

Table S4: Measured and simulated peak widths for spectrum two (taken on spot M22) of the PEG2000 sample collected using the LA (tuned) method (data plotted in Figure 2).

Mass	Measured FWHM (ns)	Simulated FWHM (ns)	Difference (ns)
1581.917	3.31	5.14	1.83
1625.944	3.61	4.94	1.33
1669.970	5.45	3.63	-1.81
1713.996	3.79	3.35	-0.44
1758.022	3.79	3.12	-0.67
1802.048	5.56	2.89	-2.67
1846.075	6.78	2.61	-4.16
1890.101	6.01	2.36	-3.64
1934.127	6.17	2.11	-4.06

1978.153	5.37	1.86	-3.52
		RMS (ns)	2.75

Table S5: Measured and simulated peak widths for spectrum two (taken on spot M21) of the PEG2000 sample collected using the LB (detuned) method (data plotted in Figure 3).

Mass	Measured FWHM (ns)	Simulated FWHM (ns)	Difference (ns)
1581.917	14.88	3.81	-11.07
1625.944	16.42	3.61	-12.81
1669.970	17.60	3.38	-14.22
1713.996	17.10	3.18	-13.92
1758.022	16.00	2.93	-13.07
1802.048	11.10	2.70	-8.40
1846.075	14.78	2.44	-12.34
1890.101	12.97	2.25	-10.72
1934.127	14.87	2.01	-12.86
1978.153	14.15	1.77	-12.38
		RMS (ns)	12.30

Adjusting the G1 Voltage to Improve the Accuracy of Simulated Peak Widths

As inferred from the RMS errors shown in Tables S4 and S5 (and visually in Figures 2 and 3 in the body of the text as the measured peak widths were consistently broader than the simulated ones), this direct approach to simulation generally did not yield accurate peak width results- and particularly for the LB (detuned) method.

The G1 voltage that gave the smallest RMS error was found by selecting two masses from the data set and running the simulation multiple times, each time making small changes to the G1 voltage as shown in Table S6. The voltage that gave the smallest difference between the measured and simulated peak widths was chosen as the new G1 voltage and is highlighted in green in Table S6.

Table S6: Two masses used to simulate flight time and peak width at different G1 voltages to determine which G1 voltage gave the smallest difference in peak width. That voltage is highlighted in green.

Mass	G1 Voltage (V)	Measured TOF (µs)	Simulated TOF (µs)	Measured FWHM (ns)	Simulated FWHM (ns)	Difference (ns)

1759.098	18900	27.649	27.731	3.98	7.36	3.38
1803.119	18900	27.989	28.076	4.04	7.23	3.18
1759.098	18950	27.649	27.737	3.98	5.34	1.36
1803.119	18950	27.989	28.081	4.04	5.01	0.97
1759.098	18955	27.649	27.737	3.98	5.09	1.11
1803.119	18955	27.989	28.082	4.04	4.84	0.80
1759.098	18960	27.649	27.738	3.98	4.89	0.91
1803.119	18960	27.989	28.082	4.04	4.61	0.56
1759.098	18965	27.649	27.739	3.98	4.61	0.63
1803.119	18965	27.989	28.083	4.04	4.45	0.41
1759.098	18970	27.649	27.739	3.98	4.42	0.43
1803.119	18970	27.989	28.083	4.04	4.19	0.15
1759.098	18975	27.649	27.740	3.98	4.23	0.25
1803.119	18975	27.989	28.084	4.04	3.96	-0.08
1759.098	18980	27.649	27.740	3.98	3.99	0.00
1803.119	18980	27.989	28.085	4.04	3.75	-0.29
1759.098	18985	27.649	27.741	3.98	3.85	-0.13
1803.119	18985	27.989	28.085	4.04	3.56	-0.48

The new G1 voltage was then used in TOFSim to simulate flight times and peak widths over the chosen mass range for each data set. That the adjusted values that best fit the measured data were all less than the values set on the instrument make sense, since the potentials felt by the ions some distance from the grid wire will be less than the potential applied to the ion optic itself. These new G1 voltage settings, as shown in Table S7, were then applied to all the spectra in the data set from each of the three different methods.

Table S7: Simulated voltage applied to G1 before the adjustment (IS2 instrumental value) and simulated G1 voltage adjusted to improve accuracy of the simulated peak widths.

Method Name	G1 Voltage Initial (kV)	G1 Voltage Adjusted (kV)
LA	19.00	18.98

LB	18.90	18.55

Table S8: Measured and simulated peak widths for the LA method spectrum two (taken on spot M22) using the new G1 voltage.

Mass	Measured FWHM (ns)	Simulated FWHM (ns)	Difference (ns)
1581.917	2.72	2.21	-0.51
1625.944	3.50	2.60	-0.91
1669.970	4.01	2.88	-1.13
1713.996	3.44	3.05	-0.39
1758.022	4.23	3.65	-0.58
1802.048	4.33	4.05	-0.28
1846.075	5.17	4.33	-0.83
1890.101	4.96	4.63	-0.33
1934.127	5.00	5.18	0.18
1978.153	4.64	5.53	0.89
		RMS (ns)	0.68

Table S9: Measured and simulated peak widths for the LB method spectrum two (taken on spot M21) using the new G1 voltage.

Mass	Measured FWHM (ns)	Simulated FWHM (ns)	Difference (ns)
1581.917	14.88	14.25	-0.64
1625.944	16.42	14.22	-2.21
1669.970	17.60	14.42	-3.18
1713.996	17.10	14.40	-2.70
1758.022	16.00	14.06	-1.94
1802.048	11.10	14.20	3.10
1846.075	14.78	13.90	-0.88
1890.101	12.97	14.05	1.07
1934.127	14.87	13.78	-1.09
1978.153	14.15	13.80	-0.35
		RMS (ns)	1.98

Statistical Analysis of Replicated Measured Data

The agreement between the measured and simulated flight times for the LB method (show in Table S10) continues to be consistent, as expected when compared to Table 5, in the main text.

Table S10: Example of measured vs. simulated flight times for the LB method spectrum 5 (taken on spot M21), where the flight times were measured three times.

Mass	Measured TOF (µs)	Simulated TOF (µs)	Difference (µs)
1581.917	26.220	26.289	-0.069
1625.944	26.578	26.652	-0.074
1669.970	26.932	27.010	-0.079
1713.996	27.280	27.363	-0.083
1758.022	27.624	27.712	-0.088
1802.048	27.965	28.056	-0.091
1846.075	28.300	28.396	-0.096
1890.101	28.633	28.731	-0.098
1934.127	28.961	29.063	-0.102
1978.153	29.285	29.391	-0.106
		RMS (µs)	0.089

The addition of the error bars to the peak width data collected using the LB method shown in Figure S3 show that the simulated and measured data are in high agreement with one another, although it does appear that the trends in peak width run in opposite directions.

Table S11: Example of measured vs. simulated peak widths for the LB method spectrum 5 (taken on spot M21), where the peak widths were measured three times.

Mass	Measured FWHM (ns)	Simulated FWHM (ns)	Difference (ns)
1581.917	4.69	5.99	-1.30
1625.944	5.14	5.87	-0.73
1669.970	5.16	5.67	-0.51
1713.996	5.04	5.50	-0.46
1758.022	5.06	5.32	-0.25

1802.048	5.00	5.09	-0.09
1846.075	5.47	4.93	0.53
1890.101	5.79	4.71	1.08
1934.127	5.25	4.46	0.79
1978.153	5.30	4.25	1.05
		RMS (ns)	0.77

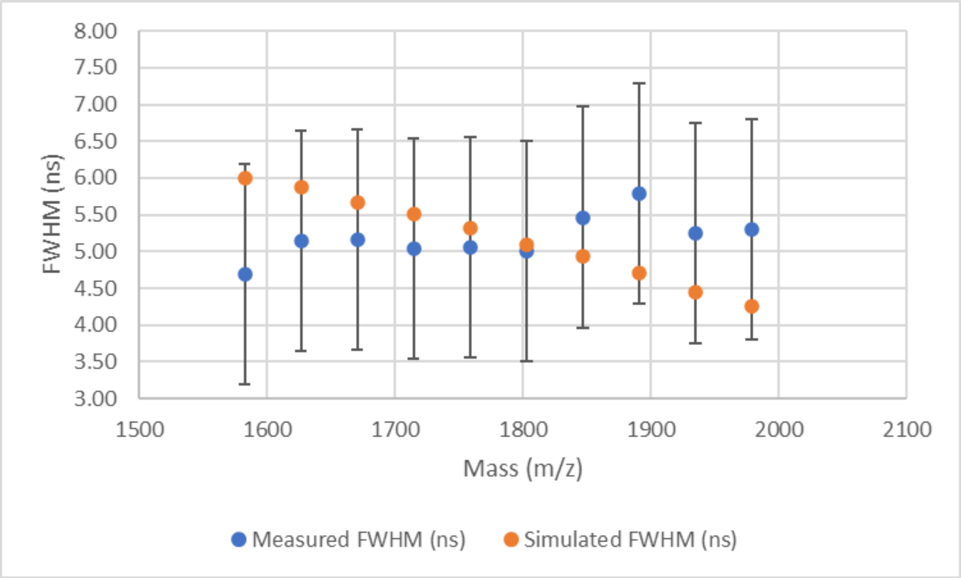

Figure S4: Plot of measured vs. simulated peak widths for the LB method spectrum 5 (taken on spot M21), where the peak widths were measured three times.

References

(S1) Fulmer, B.; Duong, A.; Palmer, H.; Owens, K. G. TOFSim: A LabView Based Time-of-Flight Mass Spectrometer Simulation", *J. Chem. Ed.*, **2024**, 101(4), 1507–1513. http://dx.doi.org/10.1021/acs.jchemed.3c00885
